# Are you a “laparoscopic surgeon”?

**DOI:** 10.4103/0972-9941.26645

**Published:** 2006-06

**Authors:** K P Balsara

**Affiliations:** GI and MAS Department, Jaslok and Bhatia Hospitals, Mumbai, India. E-mail: balsara@bom7.vsnl.net.in

Sir,

Two events in the recent past prompted me to write this letter. A young patient with polytrauma, who suffered from with recurrent bouts of small bowel obstruction, was referred to me by an orthopedic surgeon. The probable cause was a small bowel volvulus. Since the patient had minimal abdominal distension, I thought a diagnostic laparoscopy would be appropriate. Adhesions or bands could be easily tackled by laparoscopic means and if necessary, I could resort to a laparotomy. I suggested this to the referring surgeon who agreed, but asked me, “Whom would I keep standby in the event, if a laparotomy was necessary?” I was taken aback at this question, but just let it pass at that moment. Two weeks later, I was called upon to do a laparoscopic cholecystectomy for acute cholecystitis, on a surgeon's brother. When the consent form was to be signed, the same question arose. “Who will do the laparotomy, if it was needed?”

These questions made me wonder, what kind of signals have we been sending out to the medical fraternity and the lay public? I had always considered myself to be a surgeon, trained in the field of gastrointestinal surgery. The laparoscopic technique was but an extension of this training. Somewhere along the line, I got labelled as a “laparoscopic surgeon”.

At a recent congress of “laparoscopic surgeons”, I was discussing this issue with a senior surgeon from Delhi. He quite categorically said, that if he now has to convert to open surgery, he asks another surgeon to do it. His statement got me thinking. Was he right in suggesting that a surgeon doing laparoscopic surgery, not do any open work? What would be the financial and legal aspects of such an arrangement?

Financially it would impose the burden of paying two surgeons for every laparoscopic operation. Even a procedure such as a hernia would need a standby surgeon in the event of a conversion. And what about a laparoscopic- assisted operation? A right hemicolectomy is a case in point. The inside part done by the laparoscopic surgeon and the outside by the open surgeon. Sounds bizarre, but if one strictly adheres to the definition, this is what it would be.

The legal implications are even more complex. How would one frame a consent form and get the relatives to sign it? What if there was a severe morbidity or death after conversion? Who would be blamed? Would both surgeons take responsibility? I presume this happens more in realms of fairy tales, than in reality.

Calling ourselves “laparoscopic or advanced laparoscopic” surgeons gives us an air of superiority over our peers. It may also push us into doing operations which are feasible laparoscopically, though they have not been put through adequate clinical trials.[1]

J. Englebert Dunphy said, “The surgeon must be a doctor in the old- fashioned sense, an applied scientist, an artist and a minister. Because life or death often depends on the validity of surgical decisions and the surgeon's judgement must be matched by a high degree of surgical proficiency.[2] Hence, an individual approach to a patient and his disease would be better than pushing all patients to laparoscopic surgery, just because we are laparoscopic surgeons.

So should the term “laparoscopic surgeon” be abandoned? May be not, though we could put it forth more subtly. We could convey to those who refer cases to us, that we specialize in a particular branch of surgery and if appropriate, could do the operation using laparoscopic access.

Finally, what if the need arose to convert to open surgery? I believe that the primary surgeon should do this. Only in the event of real difficulty, for e.g., a bile duct or a vascular injury, should a second surgeon be called in, when the primary surgeon feels he cannot handle the situation.
